# Ignition of Free Gas in the Peritoneal Cavity: An Explosive Complication

**DOI:** 10.1155/2013/746430

**Published:** 2013-01-29

**Authors:** Aadil Mumith, Justin Thuraisingham, Sivaraman Gurunathan-Mani

**Affiliations:** ^1^Department of Trauma & Orthopaedics, St. Mary's Hospital, Parkhurst Road, Isle of Wight, Newport PO30 5TG, UK; ^2^Department of General Surgery, Dorset County Hospital, Williams Avenue, Dorset, Dorchester DT1 2JY, UK

## Abstract

We report an extremely rare event where the use of diathermy to enter the peritoneal cavity caused the free gas within it to ignite and consequently burn the surgeon during a standard right hemicolectomy procedure for a caecal perforation. This should be noted as a possible safety concern intraoperatively. We conclude that sharp dissection should always be used initially when entering the peritoneal cavity where free gas is present, cautery can subsequently be used once the free air has escaped.

## 1. Introduction

Three components are required for a fire to be produced; fuel, oxygen, and source of ignition [1]. Flammable gases are produced within the gastrointestinal tract and have been reported as the cause of theatre fires and explosions during procedures. These cases have occurred during open abdominal surgery and less invasive laparoscopic, cystoscopic, and colonoscopic procedures [2]. Some of these have proven to be fatal and hence the risk of explosion must be identified and minimised to improve patient safety when performing these common procedures. There are few documented cases of fires caused by diathermy use when entering bowel [3] however it is an extremely rare occurrence for free gas to ignite upon entering the abdominal cavity with electrocautery, we describe such a case.

## 2. Case Report

An 82-year-old gentleman presented acutely with severe onset lower abdominal pain, associated nausea and a preceding 3-day history of constipation. On examination he was afebrile, tachycardic, and his abdomen was peritonitic, a pneumoperitoneum was confirmed on an erect chest radiograph and it was decided to investigate further. Computer tomography of the abdomen revealed a caecal perforation secondary to an obstructive colonic tumour just proximal to the hepatic flexure. The patient underwent a right hemicolectomy on the emergency list shortly afterwards. 

The abdomen was prepared using a providone-iodine 10% solution. During the procedure the initial midline laparotomy skin incision was made using a scalpel followed by dissection of the subcutaneous layers and through the linea alba using monopolar diathermy set at 50/50 for coagulation and cutting, respectively. Once the peritoneal cavity was entered using cutting diathermy the free gas within the abdomen ignited forming a fireball causing partial thickness burns to the surgeon's hand. Fortunately he was able to resume the procedure after tending to his injuries. The patient remained stable and unharmed throughout the episode and had an uncomplicated recovery. 

## 3. Discussion

The combustable range of hydrogen is 4–72% and methane 5–15% [4] however they require a minimal oxygen concentration of 5% [5]. It is worth noting that oxygen concentrations in the gastrointestinal tract range from 5% in the colon to 10% in stomach however these concentrations are increased by the use of supplementary oxygen used pre- and perioperatively [6].

A distal ascending colon perforation allows gas with a highly flammable composition to leak into the peritoneal cavity. According to available data, colonic gases contain 40% of the flammable gases of the gastrointestinal tract. Human flatus contains 44% hydrogen [4] which is well within its flammable range therefore the gases within the abdominal cavity is likely to be of similar dangerous concentration in the presence of a perforation. 

The concentration of flammable gases is reduced by fasting for 12–24 hours, low residue diet, and adequate preoperative bowel washout [7]. The patient had not ingested anything for at least 12 hours prior to his presentation. Therefore suggesting that even at lower gas concentrations these risks are still present. 

To complete the fatal triad required for a fire is a source of ignition. The use of diathermy to enter the peritoneal cavity is this final component. 

This case highlights the importance of not using diathermy under any circumstance to make the initial incision when entering the peritoneal cavity whilst performing a laparotomy involving a pneumoperitoneum. All initial incisions should be made either by scalpel or dissection scissors to allow the flammable gases to escape the cavity prior to any diathermy use and hence avoiding any risk of ignition.
